# Identification of midgut membrane proteins from different instars of *Helicoverpa armigera* (Lepidoptera: Noctuidae) that bind to Cry1Ac toxin

**DOI:** 10.1371/journal.pone.0207789

**Published:** 2018-12-06

**Authors:** Igor Henrique Sena Da Silva, Isabel Goméz, Jorge Sánchez, Diana L. Martínez de Castro, Fernando Hercos Valicente, Mario Soberón, Ricardo Antonio Polanczyk, Alejandra Bravo

**Affiliations:** 1 Departamento de Fitossanidade, Faculdade de Ciências Agrárias e Veterinárias, Universidade Estadual Paulista, Jaboticabal, SP, Brazil; 2 Instituto de Biotecnología, Universidad Nacional Autónoma de México, Morelos, Mexico; 3 Embrapa Milho e Sorgo, Sete Lagoas, MG, Brazil; Institute of Plant Physiology and Ecology Shanghai Institutes for Biological Sciences, CHINA

## Abstract

*Helicoverpa armigera* is a polyphagous pest sensitive to Cry1Ac protein from *Bacillus thuringiensis* (Bt). The susceptibility of the different larval instars of *H*. *armigera* to Cry1Ac protoxin showed a significant 45-fold reduction in late instars compared to early instars. A possible hypothesis is that gut surface proteins that bind to Cry1Ac differ in both instars, although higher Cry toxin degradation in late instars could also explain the observed differences in susceptibility. Here we compared the Cry1Ac-binding proteins from second and fifth instars by pull-down assays and liquid chromatography coupled to mass spectrometry analysis (LC-MS/MS). The data show differential protein interaction patterns of Cry1Ac in the two instars analyzed. Alkaline phosphatase, and other membrane proteins, such as prohibitin and an anion selective channel protein were identified only in the second instar, suggesting that these proteins may be involved in the higher toxicity of Cry1Ac in early instars of *H*. *armigera*. Eleven Cry1Ac binindg proteins were identified exclusively in late instar larvae, like different proteases such as trypsin-like protease, azurocidin-like proteinase, and carboxypeptidase. Different aminopeptidase N isofroms were identified in both instar larvae. We compared the Cry1Ac protoxin degradation using midgut juice from late and early instars, showing that the midgut juice from late instars is more efficient to degrade Cry1Ac protoxin than that of early instars, suggesting that increased proteolytic activity on the toxin could also explain the low Cry1Ac toxicity in late instars.

## Introduction

*Bacillus thuringiensis* (Bt) is a gram-positive bacterium that in the sporulation stage, produces crystal proteins (Cry), which have selective insecticidal activity against different groups of insect pests. Bt has proven to be effective against important crop pests and against mosquitoes that are vectors of human diseases [1].

*Helicoverpa armigera*, (Lepidoptera: Noctuidae) is considered one of the most important agricultural pests in the world, affecting a wide range of different plants, among them several economically important crops such as cotton, soybean, maize, sorghum, wheat, tobacco and tomato [2]. The annual costs worldwide for controlling this pest are estimated to be more than 2 billion US dollars, excluding socio-economic and environmental costs associated with its control [3]. The insecticidal protein Cry1Ac is highly toxic for this pest and has been used worldwide in transgenic crops or spray products [4]. However, several studies reported a decrease in the susceptibility of different lepidopteran species, including *H*. *armigera*, to Cry toxins as the larval development increases [5–11].

The mode of action of Cry toxins has been shown to be a complex process involving the interaction of the toxin with different receptors in the midgut epithelium, triggering toxin oligomerization and insertion of the oligomer into the membrane, resulting in pore formation in the apical membrane of the midgut cells and the insect death [12, 13]. The most common mechanism of resistance to Cry toxins that has been observed in insect pests is linked to reduced toxin binding to brush border membrane vesicles (BBMV), including mutations inreceptor proteins genes or mutations that resulted in lower expression of Cry toxin receptors,affecting binding of the toxin [12, 14]. Different proteins have been previously described as potential Cry toxin receptors in Lepidoptera including, cadherin-like (CAD) proteins, GPI-anchored aminopeptidases (APN) and alkaline phosphatases (ALP), a 270 kDa-glycoconjugate, a 250 kDa protein named P252, an α-amylase, and recently ATP-binding cassette (ABC) transporter proteins such as ABCC2, ABCC3 and ABCA2 transporters [12, 13, 15–18]. In addition, it has been suggested that other molecules may be involved in this interaction, such as glycolipids [19]. However, it is still possible that additional proteins could be involved in the interaction with Cry1Ac toxin.

Here, we evaluated the toxicity of Cry1Ac against different larval instars of *H*. *armigera* and found significant differences in the susceptibility of the first instars versus late instar larvae. Two hypothesis could explain these differences: 1) The gut surface proteins bound by Cry1Ac change during the course of development resulting in differences in toxicity, and 2) Gut proteases increase in late instars resulting in degradation of the Cry toxin. Here we compared, by pull-down assays, the Cry1Ac binding proteins from early and late instar larvae-BBMV that may be candidates for the differences observed in toxin susceptibility among these larval stages.

## Materials and methods

### Expression and proteolytic activation of Cry1Ac toxin

Crystal inclusions of Cry1Ac toxin were produced in HD-73 strain *B*. *thuringiensis* subsp. *kurstaki*. Cells were grown for 72 h at 30°C in nutrient broth sporulation medium, as reported [20]. After complete sporulation, the crystals were solubilized in 50 mM Na_2_CO_3_-NaHCO_3_ buffer, containing 0.02% mercaptoethanol, pH 10.5, at 37°C for 2 h. The solubilized protoxins were centrifuged for 10 min at 14,000 rpm to remove the insoluble material and supernatant was stored at 4°C. For proteolytic activation of the solubilized protoxins was done using trypsin (Sigma) at 1: 32 ratio (vol: vol, trypsin: Cry1Ac) was incubated with protoxins at 37°C for 2 h. Phenylmethylsulphonyl fluoride (PMSF) (1 mM final concentration) was added to stop the proteolysis.

For pull down assays, the trypsin-activated toxins were purified by using a Mono-Q ion-exchange column (Pharmacia Biotech, Montreal, Qc, Canada). Bound toxin was eluted with a 50 to 500 mM NaCl gradient in a 20 mM Na_2_CO_3_-NaHCO_3_ buffer (pH 10.8). The protein concentration of protoxins and toxins was determined by using the Bradford method (Bio Rad, Hercules CA) and bovine serum albumin (BSA) as a standard and verified on SDS-PAGE(10% acrylamide).

### Toxicity assays

Bioassays were performed with each larval instar of *H*. *armigera* grown in artificial diet by a surface contamination method using 48 multi-well plates. The different larval instars of *H*. *armigera* were defined by measuring their cephalic capsule, as previously described [21]. At least six different concentrations of Cry1Ac solubilized crystals were assayed per larval instar. The Cry1Ac protein was poured on the diet surface and allowed to dry. One larvae of each larval instar was used per well. One plate was used per toxin concentration and at least six different toxin concentrations were assayed. These bioassays experiments were performed three times. The larval mortality was monitored after seven days of toxin administration. The mean lethal concentration (LC_50_) was estimated by Probit analysis (Polo-PC LeOra Software). The LC_50_ of the sixth instar larvae was not determined since after seven days some larvae started pupation.

### BBMV preparation

Larvae of *H*. *armigera* from a laboratory colony of the Universidade Estadual Paulista were maintained on artificial diet [22]. BBMV were prepared from dissected midgut tissue (2.5 g) of each larval instar by using the Mg-precipitation method [23] and stored at -80°C. The BBMV protein content was measured by the DC Protein dye method (Bio-Rad, Hercules, CA) using BSA as a standard. The quality of these BBMV preparations was analyzed by determining the enrichment of APN activity (as described below) in each BBMV preparation in relation to the APN activity of the homogenate. For the BBMV of first instar the enrichment was 10.2 fold higher APN activity in the BBMV vs. homogenate; for second instar was 12.7 fold; for third was 13.0 fold; for fourth instar was 5.6; for the fifth instar was 9.7 and for the sixth instar was 10.7 fold indicating good enrichment of BBM for all instars.

### ALP and APN enzymatic assays

ALP and APN enzymatic activities were determined in the BBMV samples isolated from each larval instar. APN activity was assayed using L-leucine-p-nitroanilide as substrate, and ALP activity using p-nitrophenyl phosphate as substrate [24]. Protein content was measured by the DC Protein dye method using BSA as a standard. The initial rate at 405 nm (Ultrospec II spectrophotometer; GE Healthcare) was used to calculate specific enzymatic activity of both enzymes. One unit of specific APN activity was defined as the amount of enzyme needed to catalyze the hydrolysis of 1 μmol of L-leucine-p-nitroanilide min^-1^ mg of protein^-1^ at 25°C. One unit of specific ALP activity was defined as the amount of enzyme producing 1 μmol of nitrophenol min^-1^ mg of protein^-1^ at 25°C. The nitrophenol concentration was calculated by using a standard curve of 4-nitrophenol in 0.5 mM MgCl_2_, 100 mM Tris, pH 9.5. These experiments were performed in triplicate. To analyze the significance of results, ANOVA statistical analyses were performed.

### Preparation of ALP and APN antibodies

The APN and ALP proteins from *M*. *sexta* larvae were previously cloned end heterologous expressed in *E*. *coli* cells [25, 26]. The anti-ALP or anti-APN polyclonal antibodies were raised in New Zealand white rabbits (from facilities of IBT-UNAM) after subcutaneous immunization with purified ALP or APN proteins. The rabbits were boosted three times with 1 mg of the ALP or APN proteins mixed with incomplete Freund ’s adjuvant, at 15-day intervals. Blood serum was obtained and the specificity and sensitivity of the polyclonal antisera was determined in a dot blot assay using different concentrations of ALP or APN proteins spotted on nitrocellulose strips and analyzed with different concentrations of the polyclonal anti-ALP or anti-APN antibodies and the secondary goat anti-rabbit antibody coupled to horseradish peroxidase (HRP) (diluted 1: 10,000) and visualized with Supersignal chemiluminescent substrate (Thermo Scientific, Waltham, MA) according to the instructions of the manufacturer.

### Western blots

Ten μg of BBMV from different instars of *H*. *armigera* or from third instar of *M*. *sexta* larvae were separated by SDS-PAGE and electro transferred into PVDF membranes. PVDF membranes were blocked with 5% skimmed milk in PBS buffer pH 7.4 plus 0.1% Tween 20, for 1 h at room temperature. The membranes were rinsed once with same buffer. ALP or APN proteins were detected after 1 h incubation with polyclonal anti-ALP (diluted 1: 7,500) or polyclonal anti-APN (diluted 1: 5,000) and then 1 h with goat anti-rabbit secondary antibody coupled to HRP (Santa Cruz) (diluted 1: 20,000). Western blots were visualized by incubation with Supersignal chemiluminescent substrate.

### Pull down assays

Pure Cry1Ac toxin was coupled with CNBr-activated Sepharose (GE Healthcare), according to the manufacturer’s instructions. Briefly, one mg of Cry1Ac toxin was incubated with 500 μl of CNBr agarose in 0.1 M NaHCO3, 0.5 NaCl, pH 8.3 at 4°C overnight. The non-coupled free CNBr was blocked with 0.1 M Tris-HCl at 4°C for 2 h. The noncoupled Cry1Ac protein was removed by five washes with 500 μl PBS as described by the manufacturer. BBMV prepared from the second or fifth instar larvae of *H*. *armigera* were used in the pull down assays. We used second instar since the first instar was too small to get enough midgut tissue, and we selected fifth instar since we saw that the highest APN and ALP activities were found under this developmental stage. One mg of BBMV samples were solubilized in buffer containing 1% Triton-X 100, 50 mM Na_2_HPO_4_, 50 mM NaH_2_PO_4_, 50 mM NaCl, 5 mM EGTA, 1 mM PMSF, pH 7.4 for 1 h, under agitation at 4°C. The BBMV soluble proteins were recovered by centrifugation at 50,000 rpm for 30 min to discard the non-soluble material. Soluble BBMV proteins were incubated with CNBr-Cry1Ac agarose for 1 h, under agitation at 4°C. The soluble unbound BBMV proteins were removed by an ultra-centrifugation at 30,000 rpm for 30 min at 4°C. The CNBr-Cry1Ac agarose found in the pellet, containing the bound BBMV-proteins was washed three times with 500 μl PBS supplemented with 1 M NaCl, followed by three washes with 500 μl PBS to remove all unbound proteins. High stringency of the binding buffer and washing steps was selected to reduce non-specific binding. The proteins that remained bound to the CNBr-Cry1Ac agarose were considered as Cry1Ac-binding proteins and were dissociated from the agarose by boiling for 5 min in 30 μl SDS-PAGE loading buffer (0.1 M Tris-Cl, 0.2 M DTT, 4% SDS w/v, 0.2% bromophenol blue w/v, 20% glycerol v/v, pH 6.8). The supernatant was separated by SDS-PAGE (12% acrylamide) and stained with EZ blueTM Gel Staining Reagent (Sigma Aldrich, St. Louis, MO). As negative control of this experiment, the activated CNBr agarose was coupled with 0.1 mol/L Tris-HCl amino methane buffer (pH 8.5) without Cry1Ac protein, blocked as described above, and used for incubation with same concentration of solubilized BBMV proteins.

### Identification of Cry1Ac-binding proteins by LC-MS/MS

The SDS-PAGE gels containing the Cry1Ac binding proteins from the BBMV samples of the second and fifth instar stained with EZ blue were divided into five and seven fractions, respectively. These gel fractions were cut and sent to the Proteomics Laboratory of the Institute of Biotechnology of UNAM. The sliced gel samples were reduced with dithiothreitol (DTT) (Sigma-Aldrich; St Louis, MO, USA) at 56°C for 30 min. Then, samples were alkylated with iodoacetamide (Sigma-Aldrich; St Louis, MO, USA) for 30 min in dark conditions and treated with Trypsin (Promega Sequencing Grade Modified Trypsin, Madison, WI, USA) (1: 25) at pH 8.1 at 37°C for 12 h. The peptides produced by the enzymatic cleavage were desalted with Zip Tip C18 (Millipore; Billerica, MA, USA) and five μg applied in an LC-MS/MS (Liquid Chromatography-Mass Spectrometry) system composed of an accela 600 pump (Thermo-Fisher Co.; San Jose, CA, USA) coupled to a mass spectrometer LTQ-Orbitrap Velos (Thermo-Fisher Co., San Jose, CA, USA) with nano-electrospray (ESI) ionization source. A gradient system of 5–80% solvent B (water/acetonitrile with 0.1% formic acid) was applied for 120 min using a homemade capillary column (ID 0.75 μm and 10 cm long RP-C18). The LC-MS/MS system flow was 300 nL/min. Fragmentation of the peptides was carried out by applying collision induced dissociation (CID) and high-energy collision dissociation (HCD). All spectra were acquired in positive detection mode. The execution and capture of the fragmentation data were performed dependent on the total scanning of ions according to predetermined loads with an isolation width of 3.0 (m/z), standardized collision energy of 35 arbitrary units, activation Q of 0.250, activation time of 10 ms and maximum injection time of 10 ms per micro-scan.

For data analysis, all LC-MS/MS raw data were analyzed against a Lepidoptera database constructed in house based on NCBI and Uniprot public protein data bank using the Protein Discoverer program Sequest (Thermo Fisher Scientific, San Jose, CA, USA). Sequest were searched with a fragment ion mass tolerance of 0.60 Da and a parent ion tolerance of 20 PPM. Scaffold (version Scaffold 4.8.3, Proteome Software Inc., Portland, OR) was used to validate LC-MS/MS based peptide and protein identifications. Peptide identifications were accepted if they could be established at greater than 80% probability to achieve an FDR less than 0.5% by the Peptide Prophet algorithm [27]. Protein identifications were accepted if they met the pre-established probability greater than 90%, contained at least two unique peptides identified and a coverage sequence > 4%. Protein probabilities were assigned by the Protein Prophet algorithm [28].

### Proteolytic activation of Cry1Ac, by midgut juice from different larval instar

The midgut tissue (1 g) from the different larval instars was suspended in buffer MET (300 mM Mannitol, 17 mM Tris-HCl, 5 mM EGTA, 2 mM DTT, pH 7,4) and washed three times in the same solution. The pellet was suspended in 50% dilution of buffer MET with H_2_O and stored at -70°C until used. The midgut juice was obtained after centrifugation of the midgut tissue for 10 min at 14,000 rpm at 4°C and the supernatant recovered, this procedure was repeated three times and the final supernatant was used immediately. For treatment of Cry1Ac with *H*. *armigera* midgut juice, 100 μg of Cry1Ac protoxin were incubated with a 5% dilution of the midgut juice in 20 mM Tris-HCl, pH 8.6 buffer in a final volume of 100 μl for 5 min a 4°C. The reaction was stopped with 1 mM PMSF (final concentration) and samples were analyzed in SDS-PAGE stained with Coomassie blue.

## Results

### Cry1Ac toxicity to different larval instars

We determined the toxicity of Cry1Ac toxin to each larval instar of *H*. *armigera*. Toxicity of Cry1Ac showed significant changes against larvae from the different instars. The fifth instar larvae were 45 times less sensitive to the Cry1Ac toxin than the first instar larvae (Table 1).

**Table 1:** Dose-response for Cry1Ac toxin in different larval instars of *H*. *armigera*, evaluated after 7 days by toxicity bioassay using surface contamination method.

Instar	LC_50_ (FL 95%) ^a^	Slope ± SE ^b^	Χ^2^ ^c^	Relative Toxicity
1^st^	31.1 (30.5–31.8)	0.0115 ± 0.0010	0.52	1
2^nd^	318.1 (312.6–323.8)	0.0018 ± 0.0009	0.51	10.21
3^rd^	535.6 (507.4–566.3)	0.1538 ± 0.0004	0.51	17.20
4^th^	602.9 (547.8–655.2)	0.0518 ± 0.0011	0.98	19.36
5^th^	1420.7 (1330.8–1505.3)	0.0322 ± 0.0021	0.54	45.62

^a^ Concentration killing 50% with 95% fiducial limits (FL) in parentheses, units are ng of Cry1Ac toxin per cm^2^ diet.

^b^ Slope ± standard error

^c^ Chi-square

### Expression of ALP and APN in the larvae midgut

**Fig 1:**
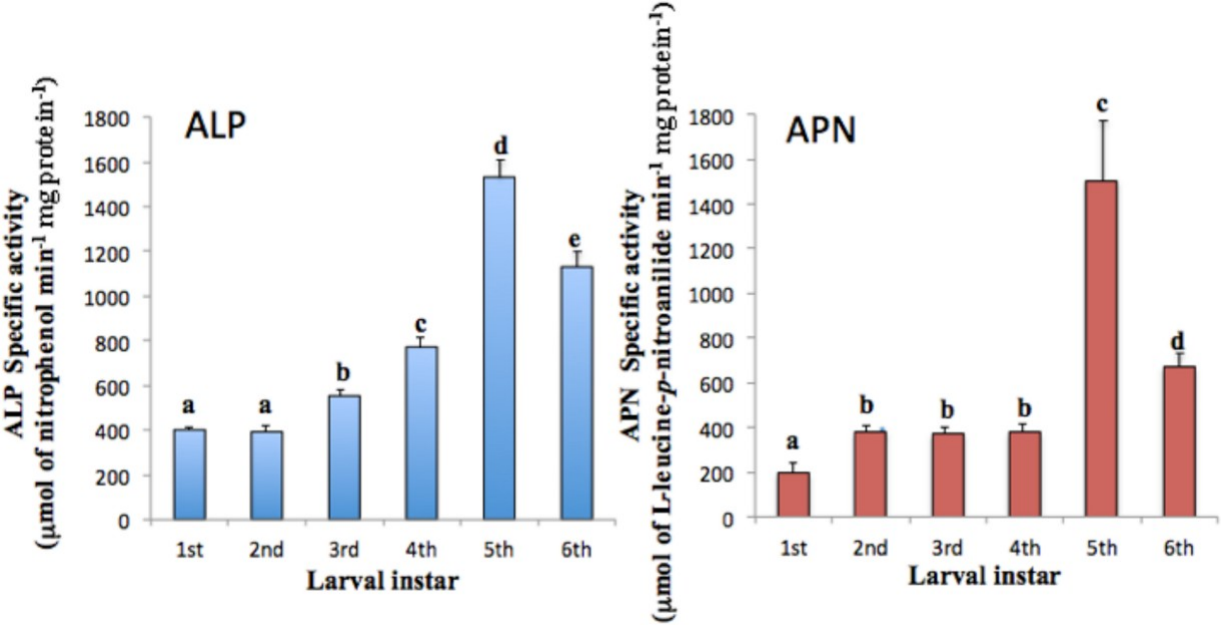
Analysis of alkaline phosphatase and aminopeptidase specific activities in BBMV isolated from different instars of *H*. *armigera*. Panel A, Specific activity of ALP. Panel B, Specific activity of APN in BBMV isolated from different larval instars of *H*. *armígera*. Letters over the bars indicate significant differences analyzed by ANOVA with significant differences P < 0.001.

**Fig 2:**
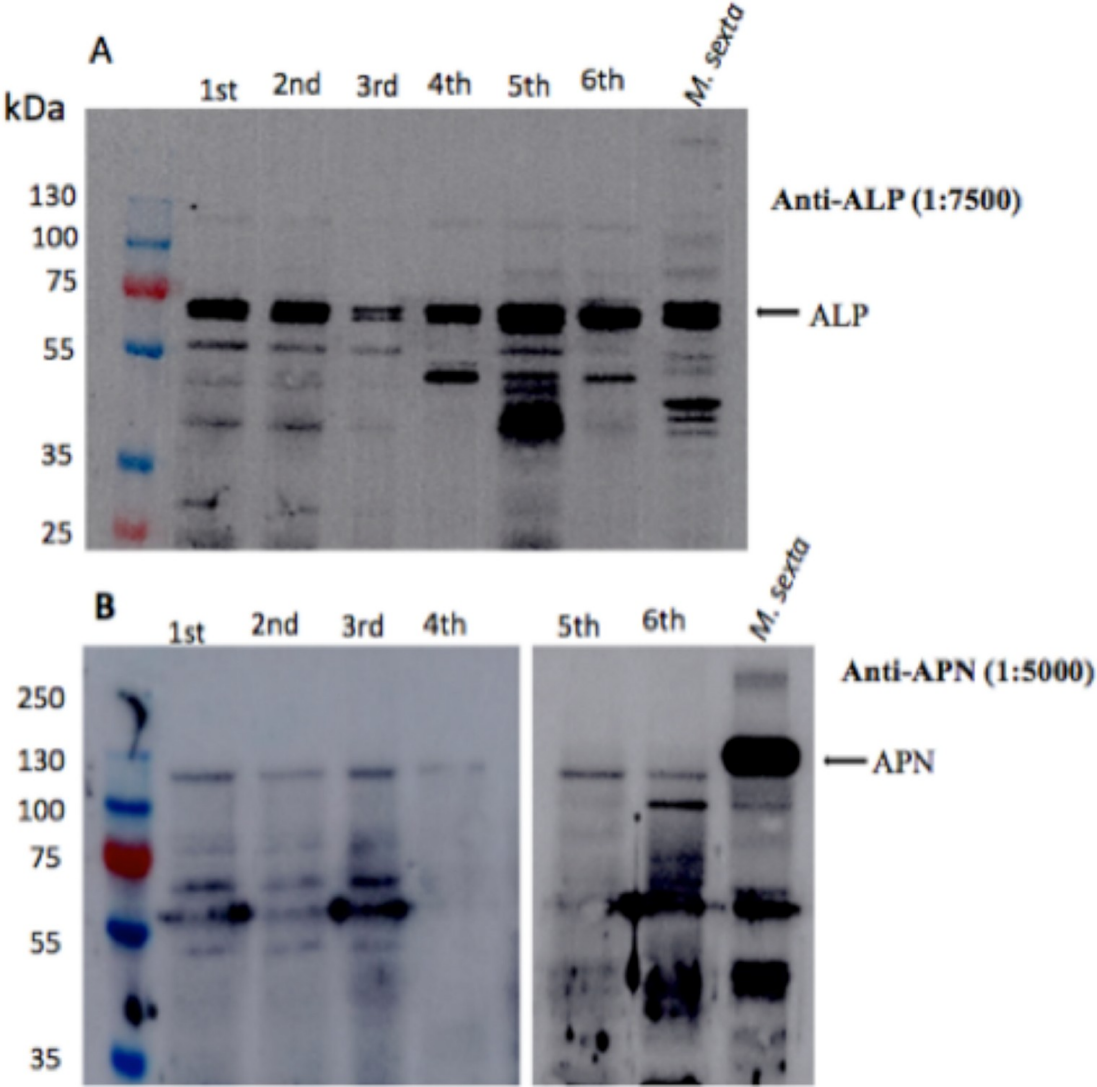
Immunodetection of ALP and APN proteins in BBMV preparations form the different instar of *H*. *armigera*. Panel A, detection of ALP protein by western blot assays using anti-ALP antibody that was raised against the ALP protein from *M*. *sexta*. Panel B, detection of APN protein by western blot assays using anti-APN antibody that was raised against the APN protein from *M*. *sexta*. The different lanes were loaded with the same concentration of protein (10 μg BBMV protein). S1 Fig shows the SDS-PAGE image of the loaded proteins stained with Coomassie Blue. Positive control was BBMV from *M*. *sexta*.

APN and ALP are two important proteins that have been recognized as Cry toxin binding proteins and have been described to be involved in larval susceptibility to Cry toxins [25, 29–32]. We decided to quantify their enzymatic activity through all developmental larval stages of *H*. *armigera*. The ALP and APN activities were present during all larval instars (Fig 1). ALP increased from the first to the fifth instar, obtaining their maxima activities at the fifth instar and then decreased in the last instar. The APN increases from the first to the second and remains relatively stable until fifth instar. To confirm the expression of ALP and APN we also performed western blots using anti-ALP and anti-APN antibodies that were raised against purified ALP and APN recombinant proteins from *M*. *sexta* as explained in materials and methods. In general the western blot assays indicated that these proteins are expressed through all instars larvae (Fig 2) and did not correlate with the observed differences in Cry1Ac susceptibility (Table 1).

In addition these data did not directly correlated with the specific activities of ALP and APN reported in Fig 1, and this may be attributable, at least partly, to a lack of specificity of the antibody as it was raised against a different *M*. *sexta* APN protein.

### Identification of Cry1Ac binding proteins by LC-MS/MS

**Fig 3:**
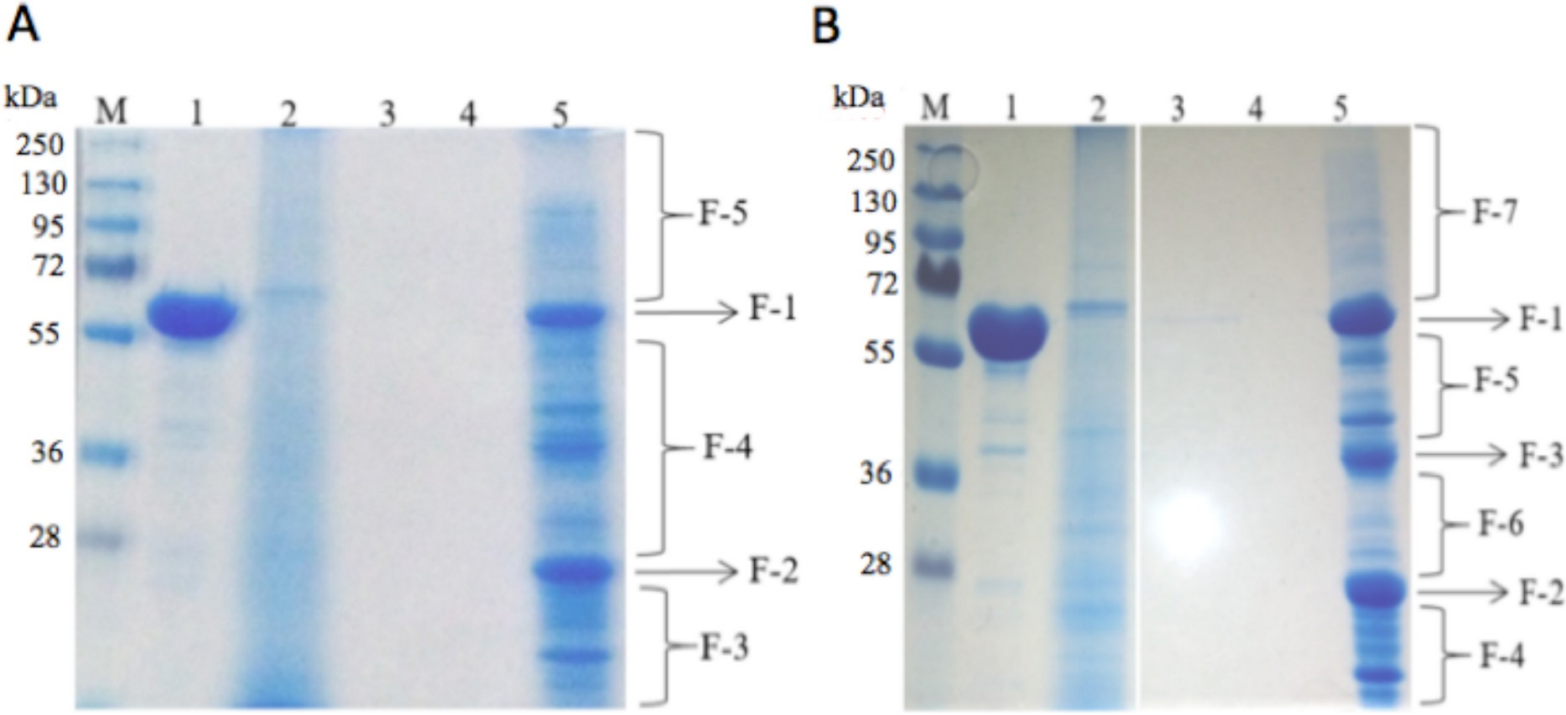
Pull down assays for identification of Cry1Ac-binding proteins in BBMV from second (Panel A) or fifth (Panel B) *H*. *armigera* larval instar. Proteins were resolved in SDS-PAGE. M, molecular marker; lanes 1, Cry1Ac toxin; lanes 2, soluble BBMV proteins from *H*. *armigera*; lanes 3 and 4, proteins found in the elution obtained after first and last wash with phosphate-buffer saline (PBS); lanes 5, BBMV proteins bound to CNBrCry1Ac. The lanes 5 containing the BBMV proteins bound to CNBr-Cry1Ac from second instar (panel A) or from fifth instar (panel B) were divided into five and seven fractions respectively. Each fraction was used for LC-MS/MS analysis.

In order to identify the proteins potentially involved in the differential toxicity of Cry1Ac toxin in young instar larvae in comparison with late instar larvae, we performed pull down assays using BBMV from second instar and compared it with BBMV from the fifth instar. As shown in Table 1, larvae from fifth instar are at least 4-fold less susceptible to Cry1Ac than larvae from second instar. Fig 3 shows the SDS-PAGE image of the BBMV proteins that bind to Cry1Ac observed after these pull-down assays with Cry1Ac toxin (lanes 5 of Fig 3). These lanes were divided into different fragments and sent for LC-MS/MS analysis. The bands of ~65 kDa in F-1 correspond to Cry1Ac toxin, that was also dissociated after boiling. In the negative control performed with blocked CNBr agarose without Cry1Ac toxin, no protein bands were observed after SDS-PAGE analysis (data not shown).

Tables 2 and 3 shows the different Cry1Ac-binding proteins that were identified in BBMV from second and fifth larval instar of *H*. *armigera*, respectively. After this screening, 25 and 33 proteins were identified bound to Cry1Ac in BBMV from second and fifth instar of *H*. *armigera*, respectively (Tables 2 and 3). Seven Cry1Ac-binding proteins were identified exclusively in BBMV from the second instar including an ion-dependent selective channel, prohibitin, lipase, ORF5 protein, RecQ helicase, ALP and an uncharacterized protein. Among the Cry1Ac-binding proteins identified in BBMV from fifth instar larvae, 11 proteins were identified exclusively in this instar including a transferrin, trypsin-like protease, azurocidin-like proteinase, carboxylesterase, alpha-amylase, chymotrypsinogen, aldehyde dehydrogenase, actin, beta-1, 3 -glucanase, carboxypeptidase, carbamoyl phosphate synthetase/aspartate. In both instars, APN isoforms 2, 3 and 4 were identified as Cry1Ac binding proteins (Tables 2 and 3).

**Table 2:** Liquid chromatography-tandem mass spectrometry assay results of binding proteins to Cry1Ac on BBMVs from *H*. *armigera* second larval instar.

Fraction	Accession number	Protein description [species] ^1^	Sequence Coverage	M.W. (kDa)	Unique peptide
F-2	D7S000_HELAM	Serine protease [Ha]	44%	25	7
	O18438_HELAM	Chymotrypsin-like protease [Ha]	23%	30	4
	Q2F5J2_BOMMO	Prohibitin protein [Bm]	16%	30	4
	B6CMF0_HELAM	Chymotrypsin [Ha]	12%	31	3
	A0A1A9FHJ3_LELAM	ATP-dependent DNA Helicase RecQ [La]	6%	69	2
	A0A023NGG2_9PICO	ORF5 protein [Se]	7%	74	2
F-3	F5BYI4_HELAM	Voltage-dependent anion-selective channel [Ha]	55%	30	14
	A3RIW4_HELAM	Aminopeptidase [Ha]	31%	98	22
	B2LRS7_HELAM	Aminopeptidase N1 [Ha]	30%	111	19
	Q962B3_HELAM	Aminopeptidase N2 [Ha]	26%	115	21
	Q7Z0W1_HELAM	Aminopeptidase N2 [Ha]	24%	115	2
	A9X7K9_HELAM	Lipase [Ha]	23%	31	4
	Q7Z266_HELAM	Aminopeptidase N3 [Ha]	21%	114	16
	Q8MU78_HELAM	Aminopeptidase N3 [Ha]	12%	114	12
	H9JUY5_BOMMO	Uncharacterized protein [Bm]	10%	34	2
	C0KH33_HELAM	Epoxide hydrolase [Ha]	6%	52	2
F-4	Q86QI6_HELAM	Aminopeptidase N4 [Ha]	24%	108	5
	Q8MU79_HELAM	Aminopeptidase N4 [Ha]	22%	108	20
	A0A0D3QSH9_ HELAM	Polycalin [Ha]	22%	102	17
	Q6R3M5_HELAM	Aminopeptidase N1 [Ha]	18%	103	4
	D5G3H6_HELAM	Aminopeptidase [Ha]	17%	75	11
	B6CMG1_HELAM	Polycalin [Ha]	11%	102	2
	A0A1B0RHM8_HELZE	Alkaline phosphatase [Hz]	8%	59	2
F5	Q962B3_HELAM	AminopeptidaseAPN2[Ha]	22%	115	18
	Q6UY55_HELAM	AminopeptidaseAPN1[Ha]	18%	113	2

Porcent Coverage > 4% (*P* < 0.05). M.W, Molecular weight (kDa).

^1^ Bm, *Bombyx mori*; Ha, *Helicoverpa armigera*; Hz, *Helicoverpa zea*; La, *Lelliottia amnigena*, Se, *Spodoptera exigua*

**Table 3:** Liquid chromatography-tandem mass spectrometry assay results of binding proteins to Cry1Ac on BBMVs from *H*. *armigera* fifth larval instar.

Fraction	Accession number		Protein description [species] ^1^	Coverage sequence	M.W. (kDa)	Unique peptide
F-2	O18439_HELAM	Diverged serine protease [Ha]		12%	27	2
F-3	D5G3H7-HELAM	Aminopeptidase [Ha]		38%	38	2
F-4	A0A1B0RHN7_HELZE		Serine protease [Hz]	22%	27	2
F-5	D5G3H7_HELAM		Aminopeptidase [Ha]	53%	38	3
	Q962B3_HELAM		Aminopeptidase N2 [Ha]	53%	115	70
	D5G3H6_ HELAM		Aminopeptidase [Ha]	49%	75	4
	B6CMF5_HELAM		Azurocidin-like serine proteinase [Ha]	45%	18	4
	B2LRS7_HELAM		Aminopeptidase N1 [Ha]	45%	111	65
	Q6R3M5_HELAM		Aminopeptidase N1 [Ha]	40%	103	2
	Q8WSZ2_HELAM		Aminopeptidase N1 [Ha]	38%	113	2
	A0A0D3QSH9_ HELAM		Polycalin [Ha]	29%	102	17
	C0KH33_HELAM		Epoxide hydrolase [Ha]	20%	52	6
	B6CMG1_HELAM		Polycalin [Ha]	19%	102	3
	Q7Z267_ HELAM		Aminopeptidase N3 [Ha]	16%	114	11
	Q86QI6_HELAM		Aminopeptidase N4 [Ha]	14%	108	8
	B1NLD_HELAM		Alpha-amylase [Ha]	13%	56	4
	Q9NHZ9_HELPN		Aminopeptidase N1 [Hp]	12%	112	4
	D2SNX0_HELVI		Aldehyde dehydrogenase [Hv]	11%	41	2
	H9JXT1_BOMMO		Uncharacterized protein [Bm]	9%	49	2
	A3RIW4_HELAM		Aminopeptidase [Ha]	8%	98	5
	B1NLE1_HELAM		Beta-1,3-glucanase [Ha]	6%	42	2
F-6	C3SBK6_HELAM		Trypsin-like protease [Ha]	25%	32	6
	B1NLE4_HELAM		Protease [Ha]	19%	28	3
	X5CT98_9NEOP		Actin [Zf]	13%	44	2
	Q6H961_HELAM		Carboxypeptidase [Ha]	6%	48	2
	H9JHG0_BOMMO		Uncharacterized protein [Bm]	5%	81	2
	Q3T905_HELZE		Carboxypeptidase [Hz]	5%	48	2
	L7QPK2_9NEOP		Carbamoylphosphate synthetase/aspartate transcarbamylase/dihydrorotase [Nm]	4%	109	2
F-7	O18438_HELAM		Chymotrypsin-like protease [Ha]	37%	30	3
	A0PGD5_HELPN		Chymotrypsinogen [Hp]	14%	30	2
	A0A067YAW9_HELAM		Transferin [Ha]	11%	83	4
	H9J9I6_BOMMO		Uncharacterized protein [Bm]	7%	80	3
	A0A127KQ69_HELAM		Carboxylesterase [Ha]	6%	79	3

Porcent Coverage > 4% (*P* < 0.05). M.W, Molecular weight (kDa)

^1^ Bm, *Bombyx mori*; Ha, *Helicoverpa armigera*; Hp, *Helicoverpa punctigera;* Hz, *Helicoverpa zea*; Hv, *Heliothis virescens*; Nm, *Nematopogon magna;* Zf, *Zigaena filipendulae*.

### Analysis of the capacity of gut extracts from different larval instars to digest Cry1Ac protoxin

**Fig 4:**
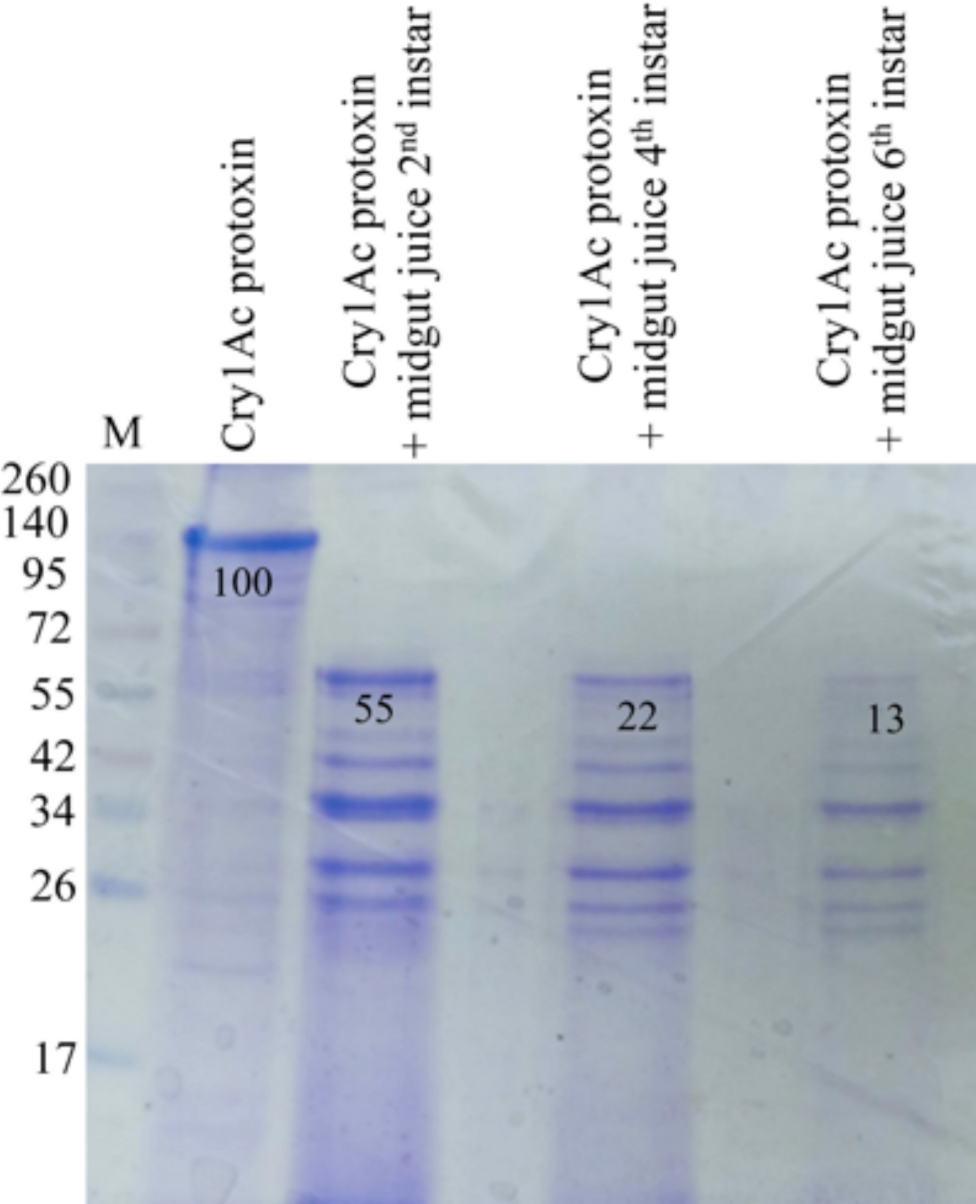
SDS-PAGE electrophoresis of Cry1Ac protoxins activated with midgut juice isolated from the midgut of different larval instars of *H*. *armigera*. The samples were analyzed by SDS-PAGE stained with Coomassie blue. Molecular masses are indicated in kDa. Numbers under the bands represent the percentage of each band on the blot calculated after densitometry analysis of the bands using ImageJ software and selecting the band of the protoxin as 100% reference.

To compare the protease activity present in the different instars of *H*. *armigera* that could digest Cry1Ac protoxin, the Cry1Ac protoxin was treated with midgut juice isolated from *H*. *armigera* from different larval instars. Fig 4 shows that treatment with midgut juice from fourth and sixth instar resulted in lower yields of the 60 kDa activated toxin indicating that the midgut juice samples from these larval-instars promote an increased degradation of the Cry1Ac toxin.

## Discussion

The Cry1Ac toxicity against *H*. *armigera* during its larval development was determined showing that there are significant changes in the susceptibility of the different instars to this toxin. The fifth instar larvae were 45 times less sensitive to the Cry1Ac toxin than the first instar larvae. The reduction in the Cry-toxin susceptibility in late instar larvae has been observed previously in other lepidopteran species. For example in *Ostrinia nubilalis* it was reported that the LC_50_ value of the fifth instar to a Dipel formulation containing Bt Berliner subsp. *kurstaki*, was 98.1-fold higher, than the first instar [6]. In *M*. *sexta* the LC_50_ of Cry1Ab was 50 times higher in the fifth instar than in the first instar [9]. In *H*. *armigera* the LC_50_ value for Cry1Ac was 16 fold higher in the second than in the first instar, while the toxicity of Cry1Aa was 200 fold higher in the second than in the first instar [10]. These differences in Cry-tolerance could be related to physiological and behavioral specificities, since first instar larvae generally ingest lower amount of food but requires much less toxin concentration to die, considering that their gut contains a lower number of cells to be disrupted.

In the case of *M*. *sexta*, the the toxicity of Cry1Ab was also observed to by substantially lower in late instars compared to first instar larvae [9], the authors proposed that a higher concentration of Cry1Ab toxin would be needed to bind to a higher number of CAD molecules in order to kill the late instar larvae since they observed an increased number of CAD receptor per midgut surface area in late instar larvae [9]. However, contradictory data were published, showing that in different lepidopteran larvae, such as *Thaumetopoea pityocampa*, *Lymantria monacha*, *Heliothis virescens*, *Spodoptera exigua* and *M*. *sexta*, the lower Cry-toxicity correlates with a decreased number of binding sites [7, 25, 29, 30, 31]. In the case of *T*. *pityocampa* and *L*. *monacha* it was shown that specific saturable binding sites for Cry1Ab were detected only in first- and second-instar larvae, not in last-instar larvae correlating with the reduction on Cry1Ab toxin susceptibility observed in the last instar larvae [7]. In the case of *M*. *sexta*, the high susceptibility to Cry1Ab observed in the first instar larvae, correlated with higher binding interaction of the Cry1Ab toxin with ALP, that was not observed in late instars [24, 25]. Also, in some cases resistance to Cry toxins have been linked to lower expression of ALP and APN proteins [29]. In addition, silencing of these receptors by RNAi has been correlated with increased tolerance to Cry toxin action [25, 30, 32, 33].

Here, we quantified the specific ALP and APN activities in the different larval instar, showing that these enzymatic activities did not correlate with the Cry1Ac susceptibility observed in the different instars. However, it is important to notice that lepidopteran larvae express multiple APN and ALP isoforms and the different Cry toxins bind specifically only to some specific APN or ALP isoforms [13], suggesting that the enzymatic activities of ALP and APN determined here, as well as their detection by western blot, correspond to all the different APN and ALP isoforms that are expressed at each instar explaining the lack of correlation between ALP and APN enzymatic activities with Cry1Ac susceptibility observed in the different instar larvae.

In order to identify the proteins involved in the interaction with Cry1Ac toxin in early and late instars, we performed Cry1Ac pull-down assays coupled with LC-MS/MS analysis using BBMV isolated from second and fifth larvae of *H*. *armigera*. Interestingly, prohibitin, an anion selective channel protein and ALP were identified as Cry1Ac-binding proteins only in the second instar. Prohibitin is a protein typically associated with lipid rafts. Lipid rafts are membrane microdomains rich in cholesterol, sphingolipids, GPI-anchored proteins and several cell-signaling receptors. It was shown, that several Cry1A receptors such as APN and ALP, were preferentially partitioned into lipid rafts and that Cry toxins insert into lipid raft of *M*. *sexta* and *H*. *virescens* [34, 35]. Prohibitin has been previously identified as Cry4Ba binding protein in *A*. *aegypti* [36] and also as Cry3Aa binding protein in *Leptinotarsa decemlineata* [37]. Silencing studies of prohibitin by RNAi in *L*. *decemlineata* showed that prohibitin is an essential protein, since its silencing affected the larval viability [37]. All these data and the fact that prohibitin is selectively found in the BBMV sample from the second instar of *H*. *armigera* could indicate that this protein may be participating in Cry1Ac toxin action in this insect pest.

To our knowledge, there are no reports showing that the anion selective channel identified here could be a Cry binding protein in any insect species. The F5BYI4_HELAM protein was described as a mitochondrial outer membrane protein, it shares 59% identity with the anion selective channel from human hVDAC3 which was also predicted to be located in the outer mitochondria membrane [38], suggesting that this protein is not likely to be exposed out side of cell in the apical membrane of midgut cells and thus could not be involved as Cry1Ac receptor.

ALP has been demonstrated to be receptor of Cry1A toxins in several insects, from different insect orders, such as Lepidoptera [29], Coleoptera [39] and Diptera [32]. Here we found that ALP protein was found in all instars but Cry1Ac pulled down ALP only from second instar BBMV suggesting that a particular ALP isoform from *H*. *armigera* was able to bind with high affinity to Cry1Ac. Previously, it was also shown that, although ALP activity is present in all developmental stages of *M*. *sexta*, the Cry1Ab toxin only binds to the ALP proteins that are expressed in young instars larvae, suggesting also that a specific ALP protein isoform from young instars participates in Cry1Ab toxin action [24, 25]. In addition, it was reported that resistance to Cry1Ac in *H*. *armigera*, correlated with a decreased transcription of a Cry1Ac-binding ALP indicating that ALP is a functional receptor of Cry1Ac in this insect species [40]. Our data support that in *H*. *armigera* a particular ALP isoform participates in Cry1Ac toxicity during the firsts instar stages.

The pull-down results shown here also identified different APN isoforms (APN1 to APN4) as Cry1Ac binding proteins in the two instars analyzed. These results indicate that APN may play a key role in the toxicity of Cry1Ac throughout the larval development of *H*. *armigera*. In fact, 38 different APNs were reported in 12 different lepidopteran species, being classified into five groups [13]. Consistently with our results APN1, APN2, APN3 and APN4 have been reported as Cry1Ac-binding proteins [41]. The different APN isoforms were identified in gel zones that are smaller than those predicted for their native size (100–120 kDa), this may have occurred due to their high abundance in the BBMV and to their high degradation susceptibility as previously reported [42]. In the case of *S*. *exigua*, it was found that a Cry1C resistant line showed lower expression of a particular APN1 protein, and it was concluded this enzyme plays a role in the resistance to Cry1Ca in this insect [30]. In the case of *Anopheles gambiae* it was shown that a specific APN2 participates in Cry11Ba toxicity [43].

Among the proteins identified exclusively in fifth instar BBMV we found some that participate in protein degradation, such as trypsin-like protease, azurocidin-like proteinase, and carboxypeptidase (Table 3). It was reported that in advanced instars of *Spodoptera littoralis* the reduced susceptibility to Cry1C was associated with protoxin inactivation by the midgut proteases. It was shown that gut juice protease profiles changed with larval development, correlating with differences in toxin degradation [5]. Here we show that midgut juice from late instars is more efficient at degrading Cry1Ac protoxin, supporting that extensive proteolysis of Cry1Ac protein could be one the reasons for the low toxicity of this toxin in late instar larvae of *H*. *armigera* (Fig 4).

The identification of the Cry1Ac-binding proteins present in the BBMV from fifth instar larvae of *H*. *armigera* was previously reported [44]. In this study, a different strategy was used, since BBMV proteins were separated by 2D-gel electrophoresis and binding of Cry1Ac was analyzed by ligand blot assays [44]. Interestingly, a different set of Cry1Ac-binding proteins was identified compared to the proteins identified here, since actin, vATPase and Hcs70 were identified [44] A possible explanation of these differences, could be due to the fact that binding assays performed in ligand blots could identified proteins that are partially unfolded due to the PAGE-SDS electrophoresis conditions, in contrast to pull down assays that detect binding proteins in non-denatured conditions. It was reported that Cry1Ac mutants that do not bind to APN in solution, can still bind to unfold APN in ligand blot assays, the authors concluded that the broader pattern of toxin-binding protein interactions could be due to the exposed peptide sequences that are found during protein denaturation. Thus, it was indicated that putative Cry toxin-binding proteins that were identified by the ligand blot assays still require to be analyzed under native conditions [45].

In a resent report, the midgut juice proteins that bind to Cry1Ab were identified from *Plutella xylostella* fourth instar larvae and from *S*. *exigua* fifth instar larvae showing that tryspsin-like proteases and Dorsal were found among the Cry1Ab binding proteins found in the midgut juice from *P*. *xylostella*, while peroxidase-C was found in the midgut juice from *S*. *exigua* [46]. It was proposed that these proteins might be sequestering the toxin and thus interfering with the insecticidal process. In addition the fact that some of these proteins are immune-related proteins may indicate that Cry toxins may alter the immune response of the insects [46], these are interesting ideas that deserved to be studies in the future.

In *H*. *armigera* it has been shown that CAD and ABCC2 transporter are functional receptors of Cry1Ac since Cry1Ac-resistant populations linked to mutations in either CAD or ABCC2 have been characterized [47, 48]. However, neither ABCC2 nor CAD proteins were detected in our pull down assays. It could be possible that the lack of detection of these proteins as Cry1Ac-binding proteins could be due to the low abundance and/or to low solubility of these proteins in the BBMV solubilization conditions used here or to their fast degradation as observed in other insects [49]. Thus, it remains to be analyzed if CAD and/or ABCC2 proteins have different expression in early and late instars that could explain in part the differences in Cry1Ac toxicity to *H*. *armigera* larvae from the different instars. Functional studies, such as RNA interference, will be carried out to determine the role of the proteins identified here in the mode of action of Cry1Ac in the different development stages of *H*. *armigera*.

## Supporting information

S1 Fig SDS-PAGE electrophoresis of BBMV isolated from the midgut tissue of different larval instars of *H*. *armigera*. The samples were analyzed by SDS-PAGE stained with Coomassie blue. Molecular masses are indicated in kDa.
(DOCX)
