# Supplementary material for: Identification of midgut membrane proteins from different instars of Helicoverpa armigera (Lepidoptera: Noctuidae) that bind to Cry1Ac toxin
Source: PLoS One. 2018 Dec 6;13(12):e0207789. doi: 10.1371/journal.pone.0207789 (PMC6283627; doi:10.1371/journal.pone.0207789)
Supplement: S1 Fig — The samples were analyzed by SDS-PAGE stained with Coomassie blue. Molecular masses are indicated in kDa. (DOCX) [file pone.0207789.s001.docx]

**Supplementary Information Identification of midgut membrane proteins from different instars of *Helicoverpa***

***armigera* (*Lepidoptera: Noctuidae*) that bind to Cry1Ac toxin**

Igor Henrique Sena Da Silva1, Isabel Goméz2, Jorge Sánchez2, Diana L. Martínez de Castro2, Fernando Hercos Valicente3, Mario Soberón2, Ricardo Antonio Polanczyk1 and Alejandra Bravo2 *****


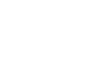

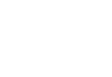

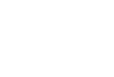

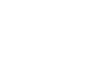

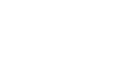

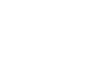

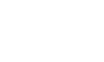


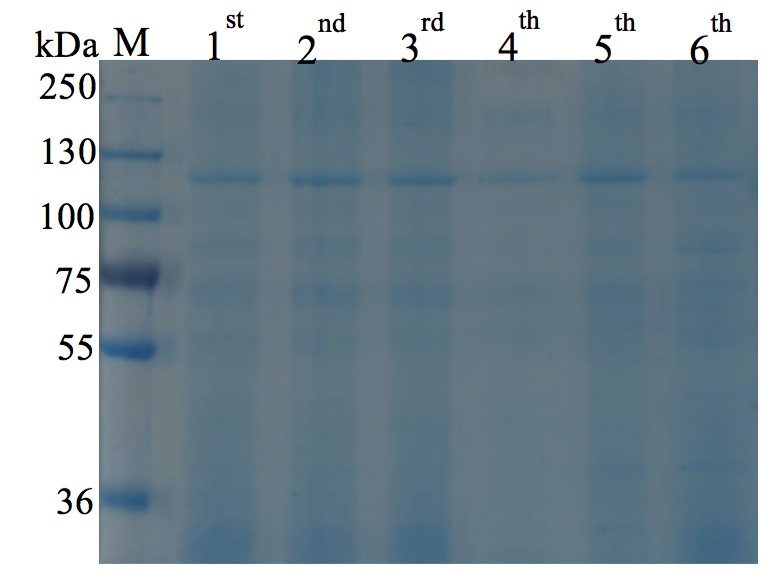

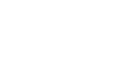

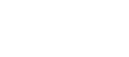

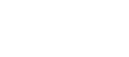

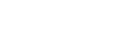

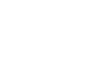

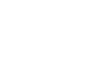

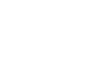


**Fig S1. SDS-PAGE electrophoresis of BBMV isolated from the midgut tissue of different larval instars of *H. armigera*.** The samples were analyzed by SDS-PAGE stained with Coomassie blue. Molecular masses are indicated in kDa.
